# Viral-Induced Enhanced Disease Illness

**DOI:** 10.3389/fmicb.2018.02991

**Published:** 2018-12-05

**Authors:** Maria K. Smatti, Asmaa A. Al Thani, Hadi M. Yassine

**Affiliations:** Biomedical Research Center, Qatar University, Doha, Qatar

**Keywords:** antibody-dependent enhancement, Fc receptors, complement, immune response, viral infections

## Abstract

Understanding immune responses to viral infections is crucial to progress in the quest for effective infection prevention and control. The host immunity involves various mechanisms to combat viral infections. Under certain circumstances, a viral infection or vaccination may result in a subverted immune system, which may lead to an exacerbated illness. Clinical evidence of enhanced illness by preexisting antibodies from vaccination, infection or maternal passive immunity is available for several viruses and is presumptively proposed for other viruses. Multiple mechanisms have been proposed to explain this phenomenon. It has been confirmed that certain infection- and/or vaccine-induced immunity could exacerbate viral infectivity in Fc receptor- or complement bearing cells- mediated mechanisms. Considering that antibody dependent enhancement (ADE) is a major obstacle in vaccine development, there are continues efforts to understand the underlying mechanisms through identification of the epitopes and antibodies responsible for disease enhancement or protection. This review discusses the recent findings on virally induced ADE, and highlights the potential mechanisms leading to this condition.

## Introduction

The relationship between virus infection and host immune response is complex. Outcomes of a virus infection are shaped by the interplay between various multilayered events of viral-host interactions. Classically, viruses initiate the cycle of infection by attachment of viral surface proteins to specific target cell receptors. Most of the viral surface proteins have extremely immunogenic structures that can bind and stimulate both innate and adaptive immune responses (Sahay et al., 2017). Neutralizing antibodies inhibit virus infection by binding to surface domains essential for virus replication such as the receptor binding site (RBS) and the fusion peptide (Taylor et al., 2015). In some circumstances, viral-induced antibodies could be detrimental, resulting in enhanced illness such as antibody dependent enhancement (ADE). ADE was first reported in 1964 by Hawkes et al. who demonstrated enhanced infectivity of a number of flaviviruses [Murray Valley encephalitis virus, West Nile virus (WNV) and Japanese encephalitis virus (JEV)] in the presence of virus-specific antisera, specifically IgG antibodies (Hawkes, 1964). Downstream studies have further explained this phenomenon.

**Figure 1:**
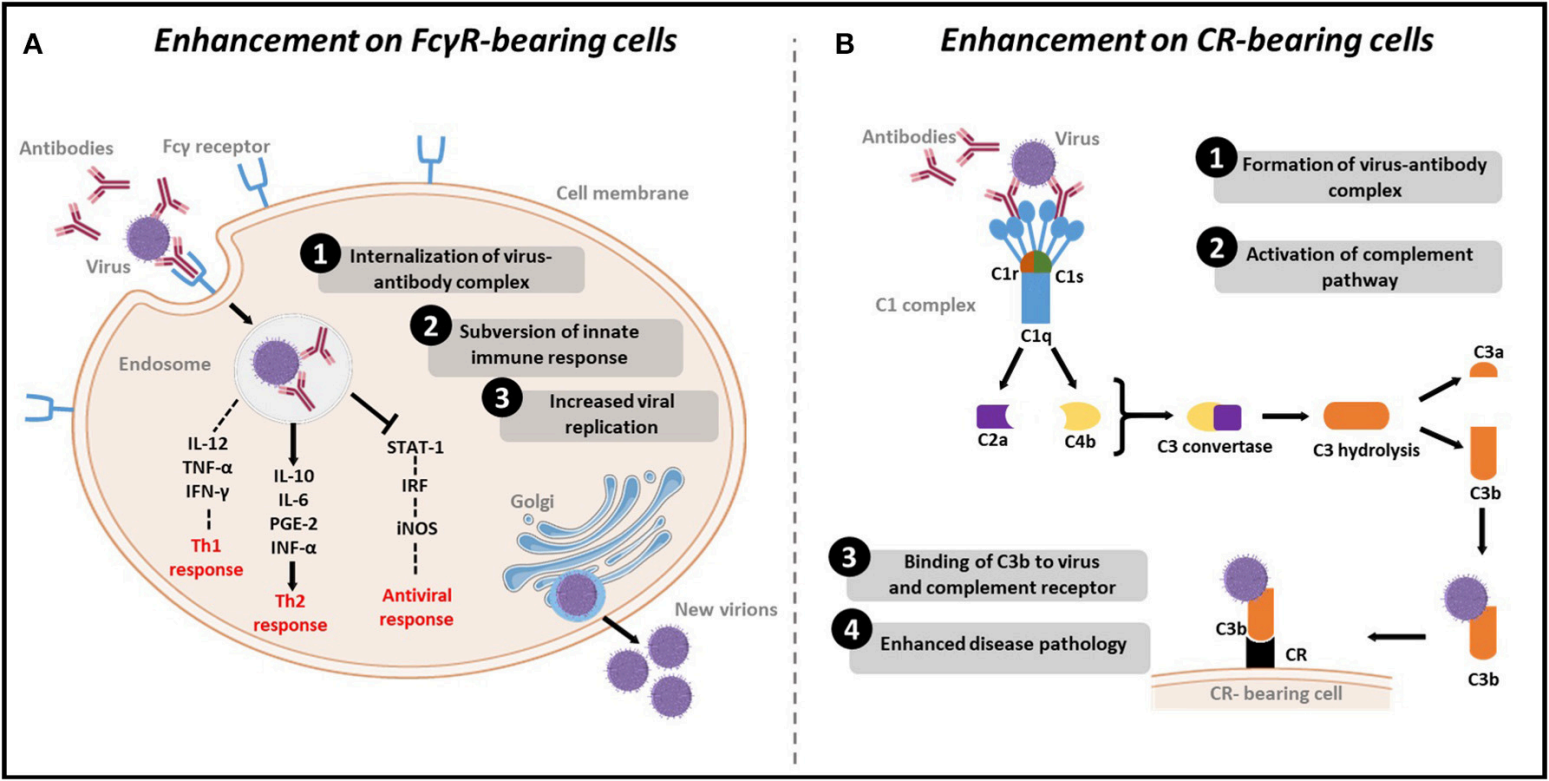
Mechanisms of ADE of viral infections. **(A)** Enhancement on FcγR bearing cells: (1) Viruses-antibody complexes are internalized to cells after antibody Fc-region binding to FcγR on the immune cells. (2) Subversion of the immune system response by reducing Th1 cytokines IL2, TNF-α and IFN-γ, increasing Th2 cytokines IL-10, IL-6, PGE-2 and INF-α, and inhibiting STAT pathway leading to decreased levels IRF and subsequent decrease in the antiviral iNOS. (3) Increased viral replication as a result of suppression of the antiviral response. **(B)** Enhancement on CR-bearing cells: (1) Formation of virus-antibody complexes. (2) This complex will activate the complement pathway by binding to C1q. Following activation, C2a and C4b proteins are recruited to produce C3 convertase, which in turn hydrolyses C3, to produce C3b. (3) C3b binds to the virus and to complement receptor (CR) on CR bearing cells. (4) Cell lysis and enhanced disease pathology.

It was shown that the binding of virions to non-neutralizing or sub-neutralizing antibodies could lead to more efficient viral uptake into the target cell in Fcγ receptor (FcγR)- (Taylor et al., 2015) or complement dependent- mediated mechanisms (Takada and Kawaoka, 2003), leading to enhanced replication and pathogenicity (Figure 1). ADE has been subsequently reported for a wide range of viruses, including flaviviruses (Dengue Halstead and O'Rourke, 1977a and Zika Bardina et al., 2017), respiratory viruses (Influenza Ochiai et al., 1988 and Respiratory Syncytial virus (RSV) Polack et al., 2002), and many others (Robinson et al., 1990a; Takada et al., 2001; Meyer et al., 2008) (Table 1).

**Table 1:** Summary of ADE observed with different viral infections.

**Virus**	**Enhanced illness manifestations**	**Host (Human/animal model/*in vitro*)**	**Proposed mechanism**	**Source of immunity/antibodies**	**Challenged with**	**Enhancing epitopes location**	**Type/class of enhancing antibodies**	**References**
Influenza	Enhanced pneumonia and lung pathology	Pigs	Anti- HA2 promote virus membrane fusion activity	Vaccination with whole inactivated H1N2 (human-like) virus vaccine (H1N2-WIV)	pH1N1	HA2 stem (amino acids 32 to 77 close to the fusion peptide)	Anti-HA2	Khurana et al., 2013
	Pneumonia - interlobular and alveolar edema- hemorrhage- peribronchiolar lymphocytic cuffing- lung lesion	Pigs	Anti- HA2 promote pro-inflammatory cytokine responses (elevated TNF-α, IL-1β, IL-6, and IL-8)	Vaccination with whole inactivated H1N2 with human like δ-cluster(δ1H1N2- WIV)	pH1N1 (A/California/04/2009)	HA2 stem close to the fusion peptide	Anti-HA2	Gauger et al., 2012
	Lung lesions, Pneumonia	Pigs	Anti- HA induced receptor mediated uptake of virus by the resident APC in the lung pulmonary alveolar macrophages (PAM)	Vaccination with whole inactivated H1N1(A/Swine/Iowa/15/1930)	H1N2 (A/Swine/Minnesota/00194/2003)	NI	IgG	Vincent et al., 2008
	Increase mortality and lung damage	Ferrets	Bias toward Th2 response (increased IFN- γ, IL10 and decreased IL-2)	Cold-adapted live attenuated vaccine (FluMist;MedImmune)	H1N1 (A/Mexico/InDRE4487/2009)	NI	NI	Kobinger et al., 2010
	Weight loss, higher lung virus titers –inflammation	Ferrets	Bias toward Th2 response (increased IL4, pro-inflammatory IL17, and IL10 cytokines)	Vaccination with 2008–09 trivalent inactivated influenza vaccine (TIV).	pH1N1 (A/Québec/144147/2009)	NI	NI	Skowronski et al., 2014
	Sever coughing, respiratory distress, severe lethargy and dyspnea- mortality	Piglets	Increased viral uptake or activation of complement fixation	Maternally derived immunity after vaccination with H1N2-δ1 whole inactivated virus (WIV) or H1N1pdm09 WIV	Heterologous strain	NI	Maternally derived Abs	Rajao et al., 2016
	Pneumonia and tracheal pathology- lymphocytic cuffing - Increased virus titer in nasal secretions	Pigs	Non-neutralizing cross-reactive antibodies form immune complexes activating inflammatory responses. (Increased IL-1β, IL-10, IL-8 and TNF- α)	Vaccination with recombinant HA protein from H1N1 (A/California/04/2009)	δ1-H1N2 (A/Swine/Minnesota/02011/2008)	NI	NI	Rajão et al., 2014
	1.4–2.5 times increased chances of developing pH1N1 illness	Humans	NI	Vaccination with 2008–09 trivalent inactivated influenza vaccine (TIV).	pH1N1	NI	NI	Skowronski et al., 2010
RSV	Airway obstruction, weight loss-pulmonary eosinophilia	Mice	Combination of Th2-biased immune response indicated by increased IL-4 and IL-13, and Th1-associated cytokine TNF-α	Vaccination with formalin-inactivated RSV (FIRSV)	RSV (A2 strain)	NI	NI	Knudson et al., 2015
	Alveolitis-associated histopathology	Cotton Rat	Low RSV F doses lead to histopathology independently of the presence of a Th1-biasing (GLA-SE) or Th2-biasing (Alum) adjuvant	Vaccination with RSV recombinant post-F or pre-F	RSV (A2 or B strains)	F protein	Anti-F	Schneider-Ohrum et al., 2017
	Severe perivascular and peribronchiolar- pneumonia and osinophilc infiltration	Mice	Lack of antibody affinity maturation	Vaccination with FIRSV	RSV	F protein	Anti-F	Delgado et al., 2009
Corona	Increased virus infectivity in FcγRII postitive, ACE2-negative human B cells	*in vitro* using human B cells	Pseudovirus-antibody complexes endocytosed via FcγRII, followed by protease cleavage of S in the endosome and S2-mediated membrane fusion in a low pH endosomal compartment	Anti-S immune serum from mice and hamsters vaccinated with recombinant native full-length S-protein trimer (triSpike)	SARS pseudotype particles (SARS-CoVpp)	S proteins	Anti-S	Kam et al., 2007
	Enhanced infection of human B cell - Promoted infection of human hematopoietic cells	*in vitro* using different immune cells	Fcγ receptor II dependent enhancement	Immune serum from mice vaccinated with whole killed SARS-CoV or recombinant SARS-CoV Spike proteins	SARS-CoVpp or Replication-competent SARS coronavirus	S proteins	Anti-S IgG	Jaume et al., 2011
	Enhanced infection of human monocyte-derived macrophages	*in vitro* using different immune cells	Internalization of immune complexes via FcγRIIs	Immune serum from mice vaccinated with recombinant SARS-CoV S proteins	SARS-CoVpp or Replication-competent SARS coronavirus	S proteins	Anti-S IgG	Yip et al., 2014
	Enhances virus infectivity and cytopathic effect of human promonocyte cells	*in vitro* using human promonocyte cells	Low concentration of anti-spike antibodies induced IL-6 and TNF-a, enhance inflammation, and caused Fcγ receptor II dependent enhancement	Anti-SARS CoV seracollected from SARS-CoV-infected patients	SARS-CoV or SARS-CoVpp	S protein (amino acids 1–460)	Anti S	Wang et al., 2014
	Lung eosinophil infiltration	Mice	Bias toward Th2 response	Vaccination with Whole inactivated virus, A recombinant DNA spike (S) protein vaccine (SV), Or virus-like particle (VLP) vaccine	SARS-CoV	NI	NI	Tseng et al., 2012
	Pneumonia, neutrophils and eosinophils infiltration - Thickening of the alveolar epithelium	Mice	Immune response to N protein cause up-regulation of both Th1 (IFN-γ, IL-2) and Th2 (IL-4, IL-5) cytokines and down-regulation of anti-inflammatory cytokines (IL-10, TGF-β)	Vaccination with recombinant vaccinia virus (VV) different combinations of SARS-CoV structural proteins	SARS-CoV	N protein	Anti N	Yasui et al., 2008
	Eosinophilic immunopathology	Mice	Immune response to N alters the host induces a Th2 skew and subsequent inflammatory pathology	Vaccination with double-inactivated SARS-CoV (DIV) vaccine	SARS-CoV	N protein	Anti N	Bolles et al., 2011
	Enhanced pulmonary inflammation	Rabbits	Complement proteins (increased C3 and C9 levels), activate immune cells and enhance inflammation	Infection with live MERS-CoV	MERS-CoV	NI	IgG	Houser et al., 2017
	Hypersensitive-type lung pathology	Mice	Bias toward Th2 response (Increased IL-5 and IL-13)	Vaccination with MERS-CoV WIV	MERS-CoV	NI	NI	Agrawal et al., 2016
DENV	Increased virus titers in FcγR- expressing cells	*in vitro* using FcγRII- expressing cells	Fcγ receptor II dependent enhancement	Sera collected from patients after DENV secondary infection	Sera collected from patients after secondary DENV infection	NI	IgG	Moi et al., 2011
	Increased viral infectivity	*in vitro* using monocytic cells	Fcγ receptor dependent enhancement	Naturally occurring human monoclonal antibodies (MAbs) isolated from subjects following vaccination or natural infection	DENV	E protein DII-FL region	Monoclonal anti-DII-FL IgG1	Smith et al., 2013
	Increased viral infectivity	*in vitro* using human myelogenous leukemia cells	Fcγ receptor dependent enhancement	Sera collected from patients after DENV depletion of serotype cross-reactive antibodies	Homotypic DENV serotype	NI	Type-specific antibodies	de Alwis et al., 2014
	Increased viral infectivity	*in vitro* using monocytic cells	Fcγ receptor dependent enhancement	Naturally occurring human monoclonal antibodies (MAbs) isolated from subjects following DENV infection	DENV 1-4	prM protein	Monoclonal anti-prM	Dejnirattisai et al., 2010
	Increased viral infectivity	*in vitro* using human monocytic cells	FcγII receptor facilitate efficient binding and entry of immature particles	Convalescent serum samples from patients infected with DENV-2.	Immature DENV-2 strain	prM protein	anti-prM	Rodenhuis-Zybert et al., 2010
	Increased viral infectivity	*in vitro* using human myelogenous leukemia cells	FcγII receptor facilitate efficient binding and entry of immature particles	Anti-prM antibody isolated from DENV-infected mice	Immature DENV-2 strain	prM protein (Amino acid 53-67)	IgG2a	Rodenhuis-Zybert et al., 2010
	Increase in viral uptake and replication in cells	*in vitro* using human myelogenous leukemia cells	Fcγ receptor dependent enhancement	Anti- prM (4D10) hybridoma generated from mouse Anti-prM (PL10) from patients sera	DENV 1-4 and Immature DENV	prM protein (amino acid 14-18)	Monoclonal anti- prM: 4D10 (IgG1) and anti- PL10	Luo et al., 2013
ZIKV	Increased viral titer	*in vitro* using human monocytic cells	NI	Human monoclonal antibodies and sera from DENV infected individuals	ZIKV strain HD78788 (African strain)	Linear fusion loop epitope (FLE)	Monoclonal anti-FLE	Dejnirattisai et al., 2016
	Enhanced ZIKV and DENV infectivity *in vitro* Lethal enhancement of DENV *in vivo*	Mice and *in vitro* using human myelogenous leukemia cells	Fcγ receptor dependent enhancement	Human monoclonal antibodies from four ZIKV-infected donors with/without previous DENV infection	ZIKV or DENV	E protein EDI/II region	Polyclonal anti EDI/II IgG	Stettler et al., 2016
	Increased mortality, morbidity, and viremia	Mice	Fcγ receptor dependent enhancement	Immune sera from individuals infected with DENV or WNV	ZIKV	E protein	Anti E IgG
	Increased viremia neutropenia, lympocytosis, hyperglycemia and pro-inflammatory response	Rhesus macaques	Fcγ receptor dependent enhancement	Infection with ZIKV (Puerto Rico strain)	DENV-2	NI	Anti DENV IgG	George et al., 2017
WNV	Increased viral infectivity	*in vitro* using human monocytic cells	Fcγ receptor dependent enhancement	Murine monoclonal antibodies produced by immunization with purified recombinant E protein or infectious WNV	WNV Reporter virus particles	E protein DIII region	Monoclonal Anti DIII	Pierson et al., 2007

*NI, not indicated*.

Virus infection or vaccination elicits different types of antibodies composed of neutralizing, non-neutralizing, enhancing, and non-enhancing antibodies (Takada et al., 2007). Virus neutralization is explained by two models: (1) the single-hit model, in which neutralization can sufficiently occur after the binding of a single antibody to a specific epitope; and (2) the multi-hit model, where neutralization requires the binding of multiple antibodies in a number that exceeds the neutralization stoichiometric threshold (Klasse and Sattentau, 2002). Recent developments in antibodies isolation and characterization, as well as epitope mapping, have led to significant progress in identifying the epitopes and antibodies responsible for either protection or disease enhancement; nonetheless, involving mechanisms are still not fully understood. Of note, understanding of ADE mechanisms will contribute to the development of safe and effective vaccines and therapies.

This review summarizes the recent findings on virally enhanced disease illness and discusses the potential underlying molecular mechanisms triggering the development of this disorder.

## Respiratory Viruses Enhanced Disease Illness

### Influenza Virus

Most of the studies concerning the humoral response to influenza viruses focus on studying the protective antibodies response to the major surface glycoprotein, hemagglutinin (HA) (Ramakrishnan et al., 2016). HA is a trimeric protein with two structurally distinct regions: the stem and the globular head (Wilson et al., 1981). Successful infection of influenza virus requires the cleavage of the HA molecule into two domains: (1) HA1, which carries the RBS; and (2) HA2, which mediates viral fusion (Steinhauer, 1999). Virus neutralization is majorly attained by antibodies targeting the HA protein, which in turn evolves recurrent amino acid mutations at the antigenic sites, in contrast to the semi-conserved stem region. Remarkably, the humoral response is also elicited against the neuraminidase (NA), which does not prevent viral infection, but may contribute to effector-mediated neutralization (Ramakrishnan et al., 2016).

The first study that reported ADE in the context of influenza infection appeared in 1980 using a rat model to assess the response to heterologous challenge after immunization (Webster and Askonas, 1980). Since then, several studies have been conducted to understand ADE of influenza *in vitro*. Ochiai et al. found that antibodies of rabbits previously exposed to influenza mediate influenza A growth in macrophages via FcγR entry and in a concentration dependent manner (Ochiai et al., 1988, 1990). Specifically, antibodies to the HA and NA proteins were shown to augment the FcγR-mediated viral uptake into antigen presenting cells (Tamura et al., 1991). These observations were further elaborated in animal models. In a study using inactivated vaccines from two swine influenza viruses (H1N1 and H1N2) to immunize pigs, protection was achieved against homologous challenge, whilst enhanced disease was observed in heterologous challenge (Vincent et al., 2008). Similar results were obtained using inactivated human-like H1N2 (delta-cluster) to immunize pigs (Gauger et al., 2011, 2012). Moreover, data from vaccinated ferret models showed that preexisting H1N1 antibodies were associated with enhanced lung disease and mortality upon infection with unmatched strain (Kobinger et al., 2010). In another study, prior exposure of ferrets to 2008-09 H1N1 trivalent inactivated vaccine (TVI) resulted in an enhanced disease after the challenge with pandemic H1N1/09 (pdm09) virus (Skowronski et al., 2014). In addition, vaccination of pigs with H1N1 pdm09 recombinant hemagglutinin (HA) subunit elicited high neutralizing antibodies to the homologous virus, but induced vaccine-associated enhanced respiratory disease (VAERD) in a heterologous virus challenge (Rajão et al., 2014). Also, in a recent study, maternally derived antibodies caused enhanced respiratory disease in piglets following heterologous influenza challenge (Rajao et al., 2016). Evidence of influenza-induced ADE was also reported in humans. Observational studies in humans showed that the 2008-09 trivalent inactivated influenza vaccine could potentially enhance pandemic H1N1 infection and illness (Janjua et al., 2010; Skowronski et al., 2010). In a direct contrast, recent work in mice to evaluate these observations showed that pre-existing non-neutralizing antibodies after administration of TIV were not associated with enhanced disease, but in fact, they were shown to promote antigen presentation and activate virus-specific CD8 T cells (Kim et al., 2016). The difference between these observations in human and mice could be attributed to several factors including: difference in antibody types (classes) between the two species, titer of antibodies in the blood, the challenge model used in mice, and many others.

Although numerous studies have reported ADE due to influenza infection or vaccination, the potential mechanisms are still not well understood. While ADE is classically explained by enhanced virus uptake in an Fc mediated mechanism, and this potential mechanism has been demonstrated in influenza infection (Ochiai et al., 1988; Tamura et al., 1991; Taylor et al., 2015), other Fc-independent mechanisms have been also suggested to cause enhancement. Using structural and viral kinetics modeling, it was shown that profusogenic stem antibodies would bind and crosslink HA2 molecules of HA, enabling them to order and orient toward the host membrane and augment viral fusion efficiency, thus, leading to enhanced virus infection (Ramakrishnan et al., 2016).

In another study, Khurana et al. characterized the antibodies associated with ADE in pigs vaccinated with whole inactivated H1N2 (human-like) vaccine and challenged with heterologous H1N1 virus (Khurana et al., 2013). Sera from immunized pigs showed high titers of cross-reactive anti-H1N1 HA antibodies that were directed to the HA2 region but not the HA1 head, and these antibodies were associated with enhanced illness. Further analysis identified that these antibodies recognize an immunodominant epitope, mapping amino acids 32–77 of pH1N1-HA2 domain. This study highlighted the role of anti-HA2 antibodies in enhancing viral fusion via Fc-independent pathway. Importantly, HA2 is known to exist in two distinct conformational forms: the pre- and post-fusion forms. Structure based analysis of HA2 revealed that residues 58–76 are located within the 34–77 epitope and are completely hidden in the pre-fusion conformation form, indicating that these enhancing antibodies are directed toward the post-fusion conformation of the HA2 (Ramakrishnan et al., 2016). In contrast, pre-fusion neutralizing stem directed antibodies act as inhibitors of viral fusion and are unlikely to cause ADE as revealed by several reports and demonstrated by Ramakrishnan et al. using mathematical model (Ramakrishnan et al., 2016). Even with the absence of measurable neutralizing activity, pre-fusion stem-directed antibodies have been shown to confer production in heterologous challenges in mice and ferrets (Yassine et al., 2015).

As observed with the respiratory syncytial virus (RSV), antibody complex formation was also proposed to lead to ADE after influenza infection (Ramakrishnan et al., 2016). In this proposed mechanism, non-neutralizing antibodies are thought to stimulate Th2 immune response, resulting in formation of immune complexes and tissue damage (Connors et al., 1994). However, as in most respiratory infections, T cell immune response to influenza is strongly biased toward Th1 cells, leading to the production of TNFα, IL-2, and IFNγ, which in turn suppress Th2 cells (Wohlleben et al., 2003; Deng et al., 2014). Hence, modulation of the immune system toward Th2 response is not supported as a mechanism of ADE in influenza.

Taken together, the current available data suggest that ADE of influenza is strain specific and can be induced primarily by non-neutralizing antibodies. Nonetheless, pre-fusion stem-directed antibodies are not likely to cause ADE. In fact, HA pre-fusion stem-only vaccine is considered a promising influenza vaccine that can induce broad protection. Moreover, the current data underscores the need for more studies and clinical trials to improve our understanding of ADE in influenza.

### Respiratory Syncytial Virus (RSV)

RSV is the major cause of hospitalization in young children. With no licensed RSV vaccine being available, RSV illness constitutes a substantial burden on health care services (Shi et al., 2017). Infection with RSV is initiated by two major surface glycoproteins, the attachment glycoprotein (G) and the fusion (F) glycoprotein, both of which are targets of neutralizing antibodies and being considered for recombinant vaccines development (McLellan et al., 2013a).

The first report of enhanced RSV disease was published in 1969. Administration of formalin- inactivated vaccine to RSV (FI-RSV) resulted in high incidence of severe illness and led to increased hospitalization in 80% among vaccinated infants, compared to only 5% of the non-immunized infants (Kim et al., 1969). Unfortunately, two vaccinated infants also died because of enhanced RSV infections (Kim et al., 1969). Using *in vitro* analysis, it was found that monoclonal antibodies directed to the two RSV surface glycoproteins but not the nucleoprotein enhanced viral infection of macrophages (Gimenez et al., 1989; Ananaba and Anderson, 1991). Further, infection enhancement was diminished by blocking Fc part of the antibody, or Fc receptor on the cells (Krilov et al., 1989). Much effort has been devoted to explain the mechanism of RSV-induced ADE using animal models. Interestingly, immunization with formalin-inactivated RSV resulted in similar responses in animal models, including monkeys (Ponnuraj et al., 2003), cotton rats (Prince et al., 1986), and mice (Waris et al., 1996). Moreover, T-helper bias toward Th2 response was detected in mice primed with inactivated virus or F glycoprotein subunit (Graham et al., 1993). Increased levels of IL-4 were associated with enhanced lung pathology, while IL-4 combined with IL-10 depletion, or depletion of CD4 cells, abrogated the enhanced pulmonary histopathology induced by RSV inactivated vaccine (Connors et al., 1992, 1994; Knudson et al., 2015). High levels of IL-13 were also observed in macaques (De Swart et al., 2002). All these observations highlighted the possible role of Th2 response in RSV-induced ADE pathology. The mechanism underlying the absence of protective antibodies against RSV after vaccination remained unexplained for long time. A widely accepted hypothesis was attributing the lack of protection to antigen alteration by formalin. Earlier work showed that the carbonyl groups on formaldehyde-inactivated RSV boost Th2 response and lead to enhanced illness (Moghaddam et al., 2006). However, other reports showed that lack of affinity maturation of the B cells following poor Toll-like receptor (TLR) activation is the reason behind eliciting non-protective antibodies (Delgado et al., 2009). This concept has been applied for other viruses such as measles, which belongs to the same family like RSV. It was found that formalin inactivated measles vaccine elicited non-protective low avidity antibodies, which resulted in atypical sever measles (Polack et al., 2003). In this case, low avidity antibodies were not able to neutralize the virus by inhibiting the binding to high affinity CD150 measles receptor. In fact, they promoted Th2 mediated immunity and led to the activation of complements and formation of immune complexes, which all in turn contributed to enhanced pathology (Delgado et al., 2009; Acosta et al., 2015). Consequently, affinity maturation can explain why ADE did not develop in children who were seropositive to RSV before vaccination. The high avidity antibodies, which were naturally developed after viral exposure, possibly conferred protection by outcompeting vaccine-induced low avidity antibodies, and thus, reduced the pathogenic B cell priming (Kim et al., 1969; Acosta et al., 2015). This also explains why ADE occurs only once in a lifetime, as naturally occurring viral infection results in the activation of new subset of B cells that mature to produce high avidity antibodies, and outcompete the vaccine-elicited pathogenic B cells (Acosta et al., 2015). This would prevent enhanced disease during reinfections and restore a healthy immune response.

It was also assumed that the RSV G protein would induce ADE in the absence of F protein. However, an RSV recombinant vaccine, which does not encode for G protein, induced a similar ADE response to the inactivated wild type virus (Johnson et al., 2004; Polack, 2007). Moreover, it was found that the conserved cysteine-rich region in the G protein has anti-inflammatory response during RSV infection through inhibiting cytokine production and antagonizing TLR (Polack et al., 2005).

Although both RSV glycoproteins (G and F) are the main targets for neutralizing antibodies (Acosta et al., 2015), the RSV F protein is more conserved and displays more neutralizing epitopes (Taleb et al., 2018). Accordingly, vaccination with F protein elicits high level of cross-reactive protection compare to G protein (Melero and Moore, 2013). The F protein in its either conformations, post-fusion (post-F), or pre-fusion (Pre-F), has been considered for development of novel vaccines and therapeutic monoclonal antibodies (Melero and Moore, 2013). Recently, it was demonstrated that FI-RSV vaccine predominantly presents the F protein in its post-fusion conformation, unlike the infectious virion, which presents both pre-F and post-F (Killikelly et al., 2016). Studies from the same group showed most of the neutralizing antibodies in human sera are directed to the pre-F protein and that immunization with pre-F elicits potent neutralizing antibodies. These data partially explain the production of non-protective antibodies following FI-RSV vaccination, where formalin inactivation not only alter virus infectivity, but also virus antigenicity. Multiple recent studies have focused on investigating the efficacy of the F protein in its either conformations as subunit vaccine. Interestingly, immunization with virus like particles (VLP) combined with adjuvants induced protective immune responses. Cimica et al. showed that immunization with pre-F or post- F VLP assembled on human metapneumovirus (hMPV) matrix protein (M) induced full protection and prevented lung disease in mice after challenge (Cimica et al., 2016). In fact, combined vaccines containing both pre- and post- F proteins induced the highest neutralization responses (Cimica et al., 2016). Further, RSV post-F subunit vaccine adjuvanted with TLR4 agonist [glucopyranosyl lipid A (GLA)] to form stable emulsion (SE) (GLA-SE) afforded robust neutralization and a Th1-directed protection in rodents (Lambert et al., 2015). Similarly, immunization of cotton rat with recombinant F in either pre or post conformation, adjuvanted with GLA-SE, elicited high neutralizing antibodies and provided protection after challenge (Schneider-Ohrum et al., 2017). Interestingly, at low vaccine doses that mimics waning immune response, lung pathology was observed independently of combining the recombinant F with a Th1 biasing (GLA-SE) or Th2 biasing (Alum) adjuvant (Schneider-Ohrum et al., 2017). These results highlight the importance of evaluating RSV vaccines over a wide dose range to assess the protective and ADE responses accurately.

There are six antigenic sites described on the F protein linked to viral neutralization. Two of which (Ø and V) are present exclusively on pre-F, and four of which (I, II, III, and IV) are present to various degrees on both conformations (Tian et al., 2017). Specifically, antigenic site Ø was found to induce higher neutralization activity than other epitopes (McLellan et al., 2013b). In a recent study, it was shown that more than 90% of RSV neutralizing antibodies in adult humans are directed to Pre-F, most of which targeted site Ø (Ngwuta et al., 2015). This highlights the importance of pre-F antigen to boost or induce functional and highly potent antibody response against RSV (Ngwuta et al., 2015). Consequently, the stabilized pre-F has been considered a promising vaccine especially for vulnerable groups including young infants, pregnant females, immunocompromised patients, and elderly. In fact, vaccination with stabilized Pre-F recombinant vaccine is being considered for pregnant women in their third trimester to boost already existing B cells and enhance transfer of maternal antibodies to newly born infants (Taleb et al., 2018). Nonetheless, a novel efficacious protective vaccine will require a careful evaluation in animals and humans in the context of enhanced disease illness.

### Coronaviruses

#### Severe Acute Respiratory Syndrome Coronavirus (SARS-CoV)

SARS-CoV first appeared in 2002 in the Guangdong province of China, followed by an epidemic in 2003, in which 26 countries were affected resulting in more than 8000 cases (WHO, 2003). Although SARS-CoV primarily infects the respiratory tract, it was found that it can directly infect and replicate in human immune cells despite the absence angiotensin I-converting enzyme 2 (ACE2; SARS receptor) on these cells (Li et al., 2004; Kuba et al., 2005; Imai et al., 2007). Antibody-mediated cell entry was suggested as a possible mechanism for SARS-CoV infection of immune cells (Taylor et al., 2015). Accordingly, Several studies have investigated the mechanisms underlying SARS-CoV mediated ADE. It was initially reported by Kam et al. that immunization with recombinant full-length SARS-CoV spike S-protein elicited protective immune response *in vivo*, but induced viral infection of human B cells *in vitro* via FcγRII-dependent and ACE2-independent pathways, suggesting novel ADE mechanism for SARS-CoV (Kam et al., 2007). Similarly, results of Jaume et al. study showed that anti-spike antibodies were able to potentiate infection of immune cells with both SARS-CoV spike-pseudotyped lentiviral particles (SARS-CoVpp) and replication-competent SARS-CoV. Likewise, this antibody-mediated infection was FcγRII dependent and ACE2 independent (Jaume et al., 2011). Another study demonstrated that enhancement of infection with replication-competent SARS-CoV as well as SARS-CoVpp in microphages was linked to anti-spike IgG, specifically through FcγRII signaling (Yip et al., 2014). Wang et al. found that ADE response is stimulated by antibodies against SARS-CoV spike proteins rather than nucleoproteins (Wang et al., 2014). It was also shown in the same study that ADE response was dependent on antibody titers, as sera with high antibodies concentration neutralized the virus, while diluted sera significantly enhanced the infection and induced more apoptosis (Wang et al., 2014). Classically, following its binding to ACE2 receptor, SARS-CoV enters susceptible cells through pH-dependent endocytosis (Yang et al., 2004). Moreover, the lysosomal cysteine protease cathepsin L was found to play a crucial role to achieve an efficient infection (Huang et al., 2006). In contrast, FcR-mediated infection occur independently of the endosomal acidic pH or the activity of cysteine proteases (Jaume et al., 2011). Unfortunately, clinical studies investigating ADE in SARS-CoV patients are limited. Several studies have reported no correlation between the clinical outcome and anti-SARS-CoV antibodies in affected individuals (Taylor et al., 2015). To the contrary, other studies correlated poor prognosis with early SARS-CoV seroconversion (Ho et al., 2005). Like the story with other viruses, vaccine-induced disease enhancement is also a concern with developing a SARS-CoV vaccine. This was reported in only small subset of SARS-CoV vaccine studies (de Wit et al., 2016). In a mouse study that investigated the role of SARS-CoV vaccine in inducing disease enhancement, Tseng et al. (2012) it was revealed that vaccines were able to protect against SARS-CoV infection, but still induced Th2 directed pulmonary immunopathology suggesting hypersensitivity to SARS-CoV components. In another study, post vaccination challenge of mice with SARS-CoV nucleocapsid protein induced sever pneumonia (Yasui et al., 2008). Likewise, double inactivated SARS-CoV vaccine in mice failed to provide complete protection and caused enhanced eosinophilic pro-inflammatory pulmonary response after infection (Bolles et al., 2011). Even with the current available data supporting the correlation between ADE and SARS-CoV preexisting antibodies, more studies are critically needed to accurately address and understand this issue in humans.

#### Middle East Respiratory Syndrome Coronavirus (MERS-CoV)

MERS-CoV was first discovered in humans in 2012. Since then, more than 2000 cases and 700 fatalities were reported worldwide (WHO, 2017). Vaccination approaches for this virus also targeted the spike protein. Some of these vaccines were evaluated in non-human models and they were shown to reduce lung pathology following challenge with MERS-CoV. Nonetheless, like other coronaviruses, vaccine-induced disease enhancement is also a concern in MERS-CoV vaccine development. MERS-CoV preexisting antibodies have been recently shown to contribute in disease enhancement. In this very recent study by Houser et al (Houser et al., 2017), researchers used New Zealand white rabbits as a model of asymptomatic MERS-CoV infection to evaluate the role of primary infection in protection from reinfection. Results showed that rabbits developed non-neutralizing antibodies against MERS viral proteins, which were not only non-protective, but also induced enhanced pulmonary inflammation, without increase in viral replication. Moreover, passive transfer of serum from infected to naïve rabbits led to disease enhancement. Further investigations revealed the contribution of complement proteins in this enhanced pathology. Specifically, an increase of C3a and C9 proteins was observed, where these proteins are responsible for the release of anaphylotoxins and recruitment of immune cells, leading to inflammation (Houser et al., 2017). In another recent study, vaccinated transgenic mice with whole inactivated MERS-CoV developed eosinophilic infiltration combined with enhanced lung disease after MERS-CoV challenge (Agrawal et al., 2016). Despite the lack of evidence of MERS-CoV-induced ADE in humans, observations from animal studies highlight the risk of potential development of enhanced disease, including sever lung pathology, in individuals who are previously infected/vaccinated with MERS-CoV. Further, the presence of non-neutralizing cross-reactivity between MERS-CoV and other circulating human coronaviruses could contribute to MER-CoV-induced ADE. Yet, this remains unstudied and requires further investigation while developing and testing coronaviruses vaccines.

## Flaviviruses Enhanced Disease Illness

### Dengue Virus

One of the most widely studied viruses in the context of ADE are dengue viruses. DENVs are group of structurally related, antigenically distinct serotypes, namely DENV1-4 (Diamond and Pierson, 2015). Infection with one serotype produces protective type-specific antibodies, but cross-reactive non-neutralizing antibodies against other subtypes that can efficiently enhance the infection (Halstead, 2014). Halstead et al. first reported enhanced DENV infection in peripheral blood mononuclear cells (PBMCs) culture obtained from immunized nonhuman primates (NHP) compared to the non-immunized ones (Halstead et al., 1973). This observation was attributed to the presence of non-neutralizing antibodies that facilitate the viral infection into the mononuclear phagocytes (Halstead et al., 1976; Halstead and O'Rourke, 1977b). Later subsequent studies revealed that cross-reactive antibodies against different DENV serotypes are predisposing factor for enhanced illness (Morens et al., 1987). DENV specific ADE was found to be correlated with increased viremia rate (Vaughn et al., 2000) and contribute to the development of severe forms of the disease including dengue hemorrhagic fever (DHF) and dengue shock syndrome (DSS) (Guzman et al., 2013). Epidemiological reports showed that individuals with preexisting dengue immunity had increased risk of developing DHF that required hospitalization (odds ratio of greater than 6.5) (Burke et al., 1988). Moreover, infants with passive-acquired immunity from DENV immune mothers tend to develop sever DHF following primary DENV infection (Halstead et al., 2002; Simmons et al., 2007). In addition, secondary heterotypic infection was strongly associated with high viral loads and sever disease (Endy et al., 2004). As with many other viruses, FcR-mediated, antibody-dependent enhancement is the main mechanism for dengue enhanced disease (Takada and Kawaoka, 2003). This mechanism has been proven through different models and experiments *in vivo* and *in vitro*. For example, the use of FcγR-expressing BHK cells showed that sera from patients after secondary infection produced 10-folds higher virus titers, compared to FcγR-negative cells and sera after primary infection (Moi et al., 2011). Complement receptors (CR) have been also shown to play a role in DENV enhanced illness. This mechanism was first described by Cardosa et al in early 1980s for another flavivirus, West Nile Virus (WNV), where the enhancement is mediated through CR3 and IgM-dependent manner (Cardosa et al., 1983). Notably, the CR mechanism of enhancement suggests that infections can spread to non-immune cells as well, since CRs are widely distributed in many cells and are not exclusively restricted to immune cells like FcγRs (Takada and Kawaoka, 2003).

The case with DENV is more complicated, not only due to the circulation of four antigenically cross-reactive serotypes, but also due to the diversity within genotypes and the generation of quasispecies during dengue infection, which may affect the humoral response and the progression of the disease in secondary infections (Kurosu, 2011; Taylor et al., 2015). In naturally occurring DENV infection, most of the produced antibodies are cross-reactive but poorly neutralizing, while serotype-specific and highly neutralizing antibodies represent only a small fraction (Fibriansah et al., 2015). Similar to natural infection, DENV live attenuated vaccines generate cross-reactive non-neutralizing antibodies leading to enhanced disease (Taylor et al., 2015). Accordingly, structural-based design of chimeric DENV vaccine that induce cross-protective neutralizing antibody response have been evaluated. The first licensed DENV vaccine, CYD-TDV, is a tetravalent live-attenuated vaccine designed of chimeras of yellow fever 17D with the four DENVs (Halstead, 2018). This vaccine was first licensed in 2015 in Mexico, and is now used in 20 countries (WHO, 2018). However, after completing phase 3 clinical evaluation, the vaccine showed to cause adverse effects in certain circumstances, resulting in more hospitalization. Accordingly, recommendations were made by the manufacturer and the WHO to vaccinate only seropositive individuals aging between 9 and 45 years in endemic areas, considering that enhanced disease can occur when vaccinating seronegative individuals (WHO, 2018).

The glycoprotein shell of the DENV consists of the envelope (E) and the membrane (M) proteins which have different conformations in the premature and mature virus, conferring a unique structure in each form (Perera and Kuhn, 2008). In the immature virus particle, precursor membrane M (prM) and E proteins form heterodimers that make trimeric spikes. During maturation, furin cleavage releases the N-terminal that contains the pr peptide of the prM protein, leaving the M as a transmembrane protein, and the proteolytic product pr peptide associated with E protein, which is found as homodimers on the virion surface (Hanley and Weaver, 2010). The E protein is composed of three domains: E domains I- III (EDI, EDII, and EDIII), and it is responsible for binding to the cellular receptors and mediating virus fusion and entry (Kuhn et al., 2002; Roehrig, 2003). It has been shown that antibodies to the E protein are the major component of DENV neutralizing humoral response (Pierson, 2010). Serotype-specific monoclonal neutralizing antibodies to the E protein have also been isolated (Shrestha et al., 2010). Several studies have characterized the neutralizing epitopes of the E protein. More than 15 structurally different epitopes on the E proteins are recognized by antibodies (Pierson, 2010). It was suggested that some of these epitopes, such as the domain II fusion loop (DII-FL), are conserved and elicit cross-reactive neutralizing antibodies to different DENVs and other flaviviruses as well (Matsui et al., 2010; Pierson, 2010; Shrestha et al., 2010). More recent studies, however, showed that these antibodies often exhibit low to moderate neutralizing activity and might result in enhanced illness (Smith et al., 2013). In contrast, one mAb recognizing the bc loop of domain II adjacent to the fusion loop (amino acids 73–79) was found to have better neutralization and cross-reactivity to all four serotypes (Smith et al., 2013). Several neutralizing human mAb were isolated form DENV patients. Notably, some of those mAb were recognizing DI and II, however, other antibodies were conformation-dependent (Sasaki et al., 2013). More recently, it was found that, unlike mouse-generated neutralizing mAb after immunization with recombinant E protein, most of the human neutralizing mAb bind to the intact virion but not to the purified recombinant protein (Gallichotte et al., 2015). It was suggested that most human neutralizing antibodies are generated against complex quaternary structures that are only assembled on the virion. This can be explained by the unique arrangement of the E proteins lying flat on the surface, which if bound to the antibodies, structural rearrangement and viral fusion is prevented (Fibriansah et al., 2015). Quaternary structure-dependent antibodies were commonly isolated (de Alwis et al., 2012), such as 1F4 and 14c10, which are sero-specific human mAb targeting the DI-II region of the E protein (Teoh et al., 2012; Fibriansah et al., 2014). 5J7 is another highly potent human mAb that was identified to bind the three functional E protein domains at the same time, which are being considered as promising targets for novel vaccine designs (Fibriansah et al., 2015). Using antibody-depletion techniques, removal of serotype cross-reactive antibodies eliminated the enhancement of heterotypic serotypes, while the enhancement of homotypic serotype was not affected, indicating that the homotypic enhancement can occur if the type-specific antibodies are present at low concentrations (de Alwis et al., 2014). Further analysis by depletion of E antibodies using recombinant E protein (rE) resulted in partial decrease in DENV enhancement illness, indicating that anti-E antibodies are partially responsible for DENV ADE (de Alwis et al., 2014). Moreover, competition studies using prM-specific Fab fragments revealed that both E and prM-specific antibodies might cause FcγR mediated ADE (de Alwis et al., 2014). Although anti-prM antibodies constitute a major component of anti-DENV humoral response, most are highly cross-reactive with low neutralizing potency even at high concentrations, thus, promote FcγR mediated ADE (Dejnirattisai et al., 2010). When cells are infected with DENV, they release high quantity of immature prM-containing virions due to the unsuccessful prM cleavage, provoking the generation of prM antibodies. These antibodies have been found to be elevated in patients with secondary infections (Lai et al., 2008). Although these immature virions are noninfectious, they restore their infectivity after interacting with prM antibodies, which in turn facilitate their binding and entry to FcγR bearing cells (Rodenhuis-Zybert et al., 2010). Interestingly, anti-prM antibodies are also shown to recognize a cryptic DI-DII junction on the E protein (Chan et al., 2012). Although this site is unavailable in functionally folded E protein, it can cause a problem when designing E protein subunit- based vaccine (Taylor et al., 2015). Additionally, a cross-reactive non-neutralizing but enhancing antibody was mapped to amino acid corresponding to residues 14–18 of the prM protein (Luo et al., 2013). In consequence, prM antibodies can contribute significantly to enhanced infection and hence, should be carefully evaluated when designing a DENV vaccine.

Structural studies have enable better understanding of the virus-antibody interaction and its impact on inducing protection or enhanced illness. Crystal structures of 2H12 Fab in complex with the highly conserved AB loop of DIII in E protein, that is poorly accessible in the mature virion, showed variable neutralizing activities in three DENV serotypes, emphasizing the role of the conformational changes in neutralization, even with conserved epitopes (Midgley et al., 2012). Therefore, it is possible that enhancing antibodies are capable of recognizing hidden epitopes that undergo conformational changes to allow antibody binding and lead to ADE (Taylor et al., 2015). More structural studies concerning the enhancing epitopes will definitely sharpen our knowledge of ADE in DENV. Importantly, most DENV studies were based on serological findings, whereas the cellular response remains less studied. In a recent study by Priyamvada et al. it has been shown that during secondary DENV infection, plasmablasts secrete high affinity and cross-reactive E specific antibodies, most of which exhibit virus neutralization *in vitro* rather than binding to recombinant envelope proteins. This again highlight the importance of virion-dependent B cell interaction and the limitation of envelope proteins binding assays (Priyamvada et al., 2016a). Further research including epitope-mapping experiments and comparative studies on plasmablasts between primary and secondary DENV infections are needed to better characterize anti-DENV broadly neutralizing antibodies, and address the role of plasmablasts in DENV cross-reactivity, serotype specificity and neutralization potency.

### Zika Virus

Zika virus (ZIKV) is a re-emerging mosquito-transmitted flavivirus. It was first discovered in Uganda in 1947 and remained restricted in parts of Africa and Asia (WHO, 2018) until 2015 where it caused a major outbreak in Brazil and rapidly spread to other American countries, and now it is endemic to several US territories (WHO, 2018). Given its wide spread, and its association to neurological disorders such as Guillain–Barre' syndrome in adults and microcephaly in newborns, ZIKV has raised a serious public health concerns worldwide. As a member of flaviviruses, ZIKV shares a considerable degree of structural similarities with other viruses from this genus including DENV and WNV (Hasan et al., 2018). Moreover, ZIKV is now circulating in many DENV and WNV endemic areas where millions of people have preexisting flaviviruses immunity (Bardina et al., 2017). Yellow fever vaccine also is administered in areas where ZIKA is circulating, such as in Central America. This highlights the possible role of ADE in ZIKA virus pathogenesis. Many recent studies have focused on studying the immunological cross-reactivity of ZIKV with other flaviviruses and its potential effect on ZIKV pathogenesis and enhanced illness. *In vitro* studies using sera from DENV seropositive individuals demonstrated the ability of DENV antibodies to enhance ZIKV infection. Several mAb derived from DENV-specific plasmablasts have been shown to cross-react with ZIKV, some of which also neutralized the virus. Both sera and mAb from DENV immune patients resulted in enhanced ZIKA infection in Fcγ receptor bearing cells (Priyamvada et al., 2016b). Antibodies to the linear DENV epitopes such as the fusion loop of the E protein were also able to drive ZIKV enhancement, as they were able to bind but not neutralize the virus (Dejnirattisai et al., 2016). Moreover, cross-reactive antibodies directed against EDI and EDII elicited by DENV or ZIKV infection, showed poor neutralization and potent enhancement of both ZIKV and DENV in cell culture, and lethal enhancement of DENV disease in mice (Stettler et al., 2016). Nevertheless, *in vitro* serological findings do not accurately reflect the actual risk of disease enhancement. The hypothesis of ADE in ZIKV after another flavivirus infection was further examined *in vivo*. ZIKV enhancement was observed in Stat2^−/−^ mice receiving human seropositive sera for DENV or WNV (Bardina et al., 2017). This was evidenced by increased mortality, morbidity, and viremia. This enhancement was mediated through IgG engagement of Fcγ receptors. However, results from this study showed also protection effect of the cross-reactive flavivirus antibodies when present at sufficient concentrations, emphasizing on the variable effects of cross-reactive flaviviruses antibodies between protecting or enhancing disease depending on their titers and surrounding physiological conditions. This study also highlighted the possible role of preexisting WNV immunity in ZIKV ADE, as a structurally related flavivirus co-circulating with ZIKV in many regions around the globe. In another recent report, prior exposure to ZIKV was also found to significantly enhance DENV infection in rhesus macaques (George et al., 2017). In contrast, Swanstrom et al. demonstrated that human mAb from DENV infected patients neutralized ZIKV in cell culture, and mediated protection in murine models (Swanstrom et al., 2016). Pantoja et al. also reported on DENV-mediated enhancement of ZIKV infection in rhesus macaques. Although their results confirmed ADE *in vitro*, there was no ZIKV enhancement in DENV immunized animals *in vivo*, rather, they found that previous exposure to DENV resulted in reduced ZIKV viremia rate compared to DENV naïve macaques (Pantoja et al., 2017). On the other hand, a study in mice indicated that ZIKV infection confers protection against WNV challenge (Vázquez-Calvo et al., 2017). The discrepancy between different studies in terms of disease enhancement or protection can be attributed to multiple factors, including: (1) The type and titer of the evaluated antibodies; (2) The strain used for infection; and (3) The used animal models. For instance, some DENV infection models are known to develop more sever neurological complications, which is not the case in humans (Zompi and Harris, 2012; Pantoja et al., 2017). While several reports have characterized the humoral response of ZIKV in correlation to preexisting flavivirus immunity, the epitopes targeted by T cells and the overall cellular response were less studied. It was found that memory T cells produced against DENV infection or vaccination recognize ZIKV conserved epitopes, leading to stronger and faster CD4 and CD8 cells responses (Grifoni et al., 2017). Interstingly, while T cell response to ZIKV targets mainly the structural proteins, it is mainly directed to nonstructural proteins in DENV infection (Grifoni et al., 2017). Finally, using trnsgenic mice, it was revealed that ZIKV-specific and ZIKV/DENV cross-reactive epitopes elicit adequate CD8 T cell responses that decreased the level of ZIKV infection (Wen et al., 2017).

### West Nile Virus

WNV is another flavivirus that has been linked to ADE since 1980s (Hawkes, 1964). The virus has three structural proteins: capsid (C), pre-membrane/membrane (prM/M) and envelope (E), and seven non-structural proteins (Colpitts et al., 2012). Human WNV infection causes subclinical febrile illness in about 80% of the cases, and leads to lethal encephalitis in < 1% of these patients. Whether this is related to ADE is still not fully known (Taylor et al., 2015). Similar to DENV disease enhancement, the main cause of ADE in WNV infection is related to the presence of sub-neutralizing concentration of antibodies, which bind to the virion and facilitate its entry to cells bearing Fc-γ receptors (Pierson et al., 2007). In addition to antibodies titer, protection or diseases enhancement by WNV mAb depends on the class and the isotype of the antibodies (Cardosa et al., 1986). Complement-mediated ADE has been also reported in WNV infection. In one study, viral infectivity of FcR-bearing cells was enhanced in the presence of antiviral monoclonal IgM antibodies, and this enhancement was blocked by anti-complement receptor type 3 (CR3) antibody but not by antibodies specific to FcR (Cardosa et al., 1983). As with other flaviviruses, the E glycoprotein is the primary target of neutralizing immune responses (Colombage et al., 1998). As previously described for DENV, domain II of the E protein encompasses epitopes near the fusion loop which elicit cross-reactive but sub-neutralizing antibodies and thus, enhance the risk of ADE (Oliphant et al., 2006). On the other hand, the most potent neutralizing antibody response to WNV infection in mice is directed to domain III of the E protein (Pierson et al., 2007). Strong neutralizing potency was demonstrated by antibodies that bind the DI–II region of E protein, such as CR4354 antibody, which was isolated from a WNV infected patient (Vogt et al., 2009). Epitopes at this region are exclusively found on the intact virion, but not on the recombinant E protein, and are composed of amino acid residues that interact with both monomers in the E dimer at the hinge region between EDI and EDII (Brandler and Tangy, 2013). Antibodies to those epitopes were found to neutralize WNV infection by neutralizing the virus in a post-attachment step, thus, preventing the rearrangement of E proteins into a fusion-active state required for the pH-induced infection of the host cell (Kaufmann et al., 2010).

## Other Viruses

ADE has also been reported for several other human and animal viruses. From the alphavirus subfamily, it was suggested early in the 1980s that sub-neutralizing titers against Semliki Forest and Sindbis viruses (SINV) could lead to antibody dependent plaque enhancement in infected macrophages (Chanas et al., 1982; Peiris and Porterfield, 1982). More recently, ADE was reported for Chikungunya virus (CHIKV) in the presence of low IgG titers elicited after single immunization by CHIKV vaccine (Hallengärd et al., 2014). Studies on Ross River virus (RRV) has also shown persistent productive infection of macrophages when using diluted sera from RRV infected patients (Linn et al., 1996). The main event of alphaviral disease enhancement involves macrophages, which are highly permissive to alphaviruses infections and are susceptible to persistent virus production (Lidbury et al., 2008).

ADE has also been recorded in Ebola virus (EBOV) infections. EBOV belongs to the *Filoviridae* family and is the causative agent of Ebola hemorrhagic fever, which is a rare but deadly disease (Burk et al., 2016). The largest Ebola outbreak occurred recently in West Africa, affected around 28,600 individuals and resulted in more than 11,300 death^1^. This massive outbreak prompted intense efforts to develop treatments and vaccines. Disease enhancement by EBOV antibodies raises the concern of an adverse vaccine effect. Several early reports showed that immunization of mice with surface glycoprotein (GP) of the Zaire strain of Ebola (ZEBOV) induces enhanced infectivity of vesicular stomatitis virus (VSV) pseudotyped with this protein *in vitro*. This enhanced activity was shown to occur through an FcR-mediated mechanism (Takada et al., 2001). Additionally, EBOV enhanced infectivity of non-monocytotic cells was found to occur by a different mechanism that requires the involvement of complement factor 1 (C1). In this case, two or more IgG molecules bind to specific epitopes in close proximity, permitting the binding of C1q to the Fc portion of the antibodies and resulting in a complex formation that enhances virus-cell interactions (Takada and Kawaoka, 2003). Subsequent studies have showed that ADE of EBOV GP was strain specific and correlated with IgG2, IgG3 and IgM but not the IgG1 class of antibodies, which has low affinity to C1q (Takada et al., 2007). A recent work by Furuyama et al. investigating the molecular mechanism of EBOV ADE highlighted the involvement of Scr family protein tyrosine kinases (PTKs) in the Fcγ-receptor IIa (FcγRIIa)-mediated ADE (Furuyama et al., 2016). They found that antibody-virus complexes bind to the cell surface FcγRIIa and trigger the Src signaling pathway, which in turn contributes to facilitating the viral entry into in human leukemia FcγRIIa-expressing cells. Additionally, specific epitopes on ZEBOV GP were found to enhance the infection. This was confirmed by the use of mouse antisera to the chimeric ZEBOV GP lacking the ADE epitopes, where less ADE activity was produced compared to the mouse antisera against the wild-type ZEBOV GP (Takada et al., 2007). Although some mAb to ZEBOV GP have shown protective effect from lethal infection in mice (Qiu et al., 2011), enhanced virus infectivity generated by other anti-GP antibodies has raised concerns of using this protein as a vaccine candidate (Taylor et al., 2015). Recently, a study investigated the ability of mAbs obtained from filovirus human survivors to induce ADE (Kuzmina et al., 2018). Results showed that at sub-neutralizing concentration, all mAbs potentiate ADE regardless of particular epitope specificity, neutralizing capacity, or the subclass. Moreover, when Fcγ receptors were blocked, or Fc domain was mutated, a reduced, yet not abolished ADE was observed with high-affinity binding antibodies. Nevertheless, low-affinity binding antibodies were still able to cause ADE. This can explain the previous failure of EBOV therapeutic attempts when insufficient doses of passively transferred antibodies were administered (Kuzmina et al., 2018). On the other hand, the use of high concentrations of monoclonal or polyclonal antibodies showed improved efficacy and absence of ADE in animal models and human volunteers (Kudoyarova-Zubavichene et al., 1999; Corti et al., 2016). Currently, there is no approved vaccine for EBOV, although many promising vaccine candidates are in clinical trials. This includes vector-based vaccines, such the replicating vesicular stomatitis virus (rVSV) and the replication-defective chimpanzee adenovirus 3 (ChAd3), DNA vaccines, and subunit vaccines (Ohimain, 2016; Pavot, 2016). Importantly, in light of previously observed EBOV ADE, which is a complex process affected by the different immunization strategies and may lead to extreme virulence, cautions should be taken in designing and developing a new Ebola vaccine. In addition, the vaccine must be carefully tested over a wide dose range in order to define the protective dose that can overcome ADE.

Similar to Ebola virus, complement-mediated and FcγRs-mediated ADE were reported with in human immunodeficiency virus (HIV) infections. It was observed that HIV seropositive sera, which typically have low neutralization activity, have the ability to enhance virus infectivity in cell culture (Robinson et al., 1987). Specifically, human monoclonal antibodies V10-9, N2-4, and 120-16, which are directed to the transmembrane glycoprotein 41 (gp41), enhanced infectivity of HIV-1 *in vitro* (Robinson et al., 1990a). It was then documented that complement receptor 2 (CR2) bearing T cells are susceptible to enhanced infectivity by HIV seropositive sera, concluding that CR2 and CD4 are essential for HIV complement dependent enhancement (Robinson et al., 1990b). On the other hand, FcR dependent enhancement was observed in macrophages and CD4 T cells, which was specifically mediated by FcRIII (Homsy et al., 1989). The entry of HIV to CD4 cells is mediated by the conformational changes that occur after the interaction between the HIV envelope protein and CD4, and then chemokine receptor (such as CCR5) on the cell surface. This induces additional conformational changes in gp120 and facilitates its fusion with cell membrane and entry of the viral capsid into the target cell (Guillon et al., 2002). Therefore, independent from the neutralization capability, antibodies to HIV can enhance the infection by producing conformational changes to the gp120 (Shmelkov et al., 2014). Although the idea of HIV ADE has been proposed decades ago, shortly after the first isolation of the virus in 1984, it has been ignored in HIV research for long time until enhanced illness was observed in HIV clinical trials (Shmelkov et al., 2014). Enhanced disease was reported in the RV144 HIV vaccine clinical trial in 2012 and was attributed to non-neutralizing envelope-specific plasma IgA antibodies (Haynes et al., 2012). Moreover, evaluation of AIDSVAX vaccine (Genentech, U.S.), showed disease enhancement as well. Statistical analysis of AIDSVAX clinical trials indicated that low antibody response to the recombinant glycoprotein 120 (rgp120) increased the rate of HIV infection compared to the control group (Gilbert et al., 2005; Shmelkov et al., 2014). Significant efforts have been made to identify T- and B- cells protecting and enhancing epitopes. HIV broadly neutralizing antibodies (bNAb), which are produced in very low number of the infected individuals, typically target conformational epitopes that are masked by glycosylation or not well exposed to the immune system. In contrast, epitopes targeted by T cells are short peptides and can be used to design a vaccine free of non-protective epitopes (Fischer et al., 2007; McCoy and Burton, 2017). Accordingly, an effective HIV vaccine should be designed to contain both, conserved T cell epitopes, as well as selected B cell epitopes that are capable of strictly eliciting bNAb, antibody-dependent cellular cytotoxicity (ADCC) and Antibody-dependent cell-mediated virus inhibition (ADCVI) rather than harmful antibody responses (Sahay et al., 2017).

Reports has also revealed ADE in herpesviruses including Epstein-Barr virus and herpes simplex virus, foot-and-mouth disease virus (Baxt and Mason, 1995), measles virus (Huisman et al., 2009), rabies virus (Porterfield, 1981), and several others (de Swart et al., 2007). Antibodies to enhancing epitopes were also described for number of other viruses. Examples of these are: mAb to hepatitis C virus E2 glycoprotein elicited after week immunization (Meyer et al., 2008), as well as mAb to the nucleocapsid (N) and glycoprotein 5 (GP5) of porcine reproductive and respiratory syndrome (PRRS) virus (Cancel-Tirado et al., 2004). Table 1 summarizes ADE observed with different viral infections.

Recent studies have shed some light on viral-induced ADE at the molecular level despite the limited progress. In addition to viral and host genetic factors, intracellular signaling was found to play a crucial role in ADE pathogenesis. Modulation of cell signaling during ADE has a remarkable effect considering its impact on different defense pathways. Classically, ADE occur through two distinct mechanisms: intrinsic and extrinsic pathways (Taylor et al., 2015). Extrinsic ADE involves facilitating virus entry and infection into the cells. As discussed earlier in this review, this mechanism is mediated by enhancing antibodies that increase virus uptake of through Fc-receptors or complement receptors. On the other hand, intrinsic ADE is the mechanism by which the innate and adaptive intracellular antiviral responses are altered, leading to increased virus production and consequently high viral loads (Flipse et al., 2013). A more favorable environment for virus survival is attained through the suppression of essential antiviral genes expression (Thomas et al., 2006). Particularly, it was found that ADE diminish antiviral responses at the transcriptional level. Mechanistic studies have shown that ADE affects the lipopolysaccharide (LPS)-induced antiviral activity in macrophages by specifically targeting the transcription and translation of essential antiviral factors, resulting in uncontrolled viral replication (Lidbury and Mahalingam, 2000). For instance, analysis of RRV-infected macrophages at the RNA level showed that enhanced infection was associated with disrupted antiviral transcription factor complexes, such as tumor necrosis factor alpha (TNF-alpha), nitric-oxide synthase 2 (NOS2), IFN regulatory factor 1 (IRF-1), as well as an increase of IL10 expression (Mahalingam and Lidbury, 2002). It was also suggested that IL10 downregulates Th1 and inflammatory cytokines (Mahalingam and Lidbury, 2002), leading toward increased viral replication (Figure 1). This observation indicates that alteration of intracellular signaling during ADE does not only suppress immune defense genes, but also enhance the expression of host genes that can contribute to virus survival (Thomas et al., 2006). Moreover, in a recent 2018 study, high throughput sequencing of miRNA from human PBMCs following DENV-3 and DENV-3 ADE infections was performed (Jiang and Sun, 2018). Results revealed that ADE was associated with altered miRNA expression profile, highlighting the role of miRNAs in modulating immune response. Nonetheless, future studies are absolutely needed to describe the exact role of miRNAs in ADE.

Virally induced enhanced diseases can also be influenced by several host genetic factors. Interestingly, a study by Boonnak et al. showed that single nucleotide polymorphisms (SNPs) in the IL10 promoter region affected the expression levels of ADE- associated IL10 (Boonnak et al., 2011). Individuals with the homozygous GCC haplotype were presented with the highest level of IL10, followed by ACC and ATA haplotypes. This study highlights the significance of genetic susceptibility of infectious diseases, and thus, the importance of identifying host genetic factors (including genes polymorphisms) related to innate and adaptive immune responses against viral infection. This includes but not limited to: cellular receptors genes such as FcγR and MHC, cytokine gens, as well as complement pathways genes.

**Figure 2:**
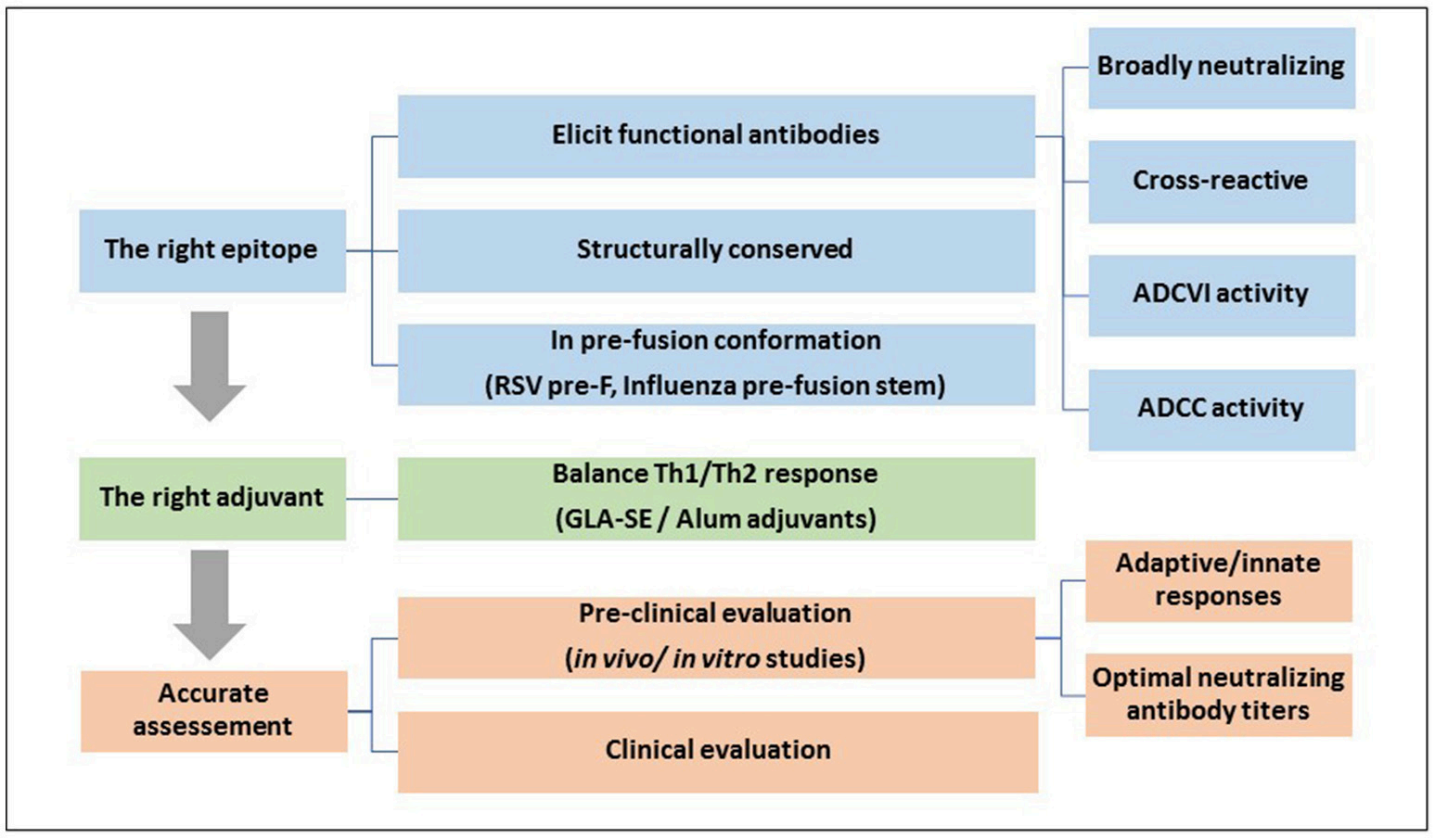
A general strategy for designing an efficacious vaccine that overcomes ADE requires: 1, Identifying the right epitope that elicits functional protective antibodies: An effective vaccine should be designed using conserved and functional epitopes, which usually exist in the pre-fusion conformation of the surface glycoproteins; 2, Selection of the right adjuvant that can direct the immune system toward a balanced Th1/Th2 response; and 3, A carful and accurate assessment of the efficacy and safety of the vaccine at different doses in pre-clinical and clinical trials.

The ADE phenomenon in different virus infections was and still an area of intensive study, especially while developing new vaccines. Any vaccine that fails to produce broad protective antibodies could lead to adverse effects by rendering vaccinated individuals more susceptible to infection as demonstrated in previous clinical trials. A successful vaccine approach relies on the design of novel immunogen that can induce a balanced and protective immunity. It is now suggested that efficacious vaccines should strictly target protective epitopes (B- and T- cell), which are able to elicit functional immune responses, such as bNAb, ADCC and ADCVI (Sahay et al., 2017). Moreover, selection of appropriate adjuvants affects vaccine immunogenicity considerably and thus, affects vaccine efficacy. Nonetheless, even when selecting the appropriate antigen and adjuvant, ADE remains a concern when neutralizing antibodies are elicited at sub-neutralizing doses. Consequently, evaluating vaccines in terms of protective and enhancing doses are crucially needed before vaccine administration (Figure 2).

## Conclusion

Virus pathogenesis and host immunity relationship is controlled by multiple factors. ADE is a complex disorder that may lead to extreme virulence for many viruses. Considering the involvement of wide number of viruses in ADE, various mechanisms were proposed to explain this phenomenon. Current observations suggest that ADE is primarily induced by non-neutralizing antibodies, via FcγR (Taylor et al., 2015), or complement dependent pathway (Takada and Kawaoka, 2003). During the course of infection, different types of antibodies are produced representing mixture of neutralizing, enhancing, and non-neutralizing antibodies (Takada et al., 2007). Virus enhancement or neutralization depend on multiple factors, including antibodies type and class, antibodies titers, strain of the virus, as well as the presence of certain complement molecules. Surface proteins are the main antigenic determinants of antibody enhancing response, such as the HA of influenza (Ramakrishnan et al., 2016), G protein of RSV, spike S protein in SARS (Kam et al., 2007), E glycoprotein of flaviviruses (Bardina et al., 2017), transmembrane GP of HIV (Robinson et al., 1990a), GP of Ebola virus (Takada et al., 2001), and E2 glycoprotein of HCV (Meyer et al., 2008).

Numerous studies have characterized the host factors (B and T cellular responses) and viral factors (targeted epitopes) responsible for disease protection/enhancement. In the context of vaccine design, it is important to ensure that all vaccine are tested at different doses to ensure the elicitation of optimal titers of neutralizing antibodies. Better understanding of viral-host interplay in the context of enhanced disease illness would greatly improve the development of highly safe and effective vaccine and therapeutics.

## Author Contributions

HY and MS designed and wrote the first draft of the manuscript. AA proofread and revised the manuscript.

### Conflict of Interest Statement

The authors declare that the research was conducted in the absence of any commercial or financial relationships that could be construed as a potential conflict of interest.

## Footnotes

^1^CDC. *Ebola (Ebola Virus Disease)*. Available online at: https://www.cdc.gov/vhf/ebola/index.html.
